# PLG nanoparticles target fibroblasts and MARCO^+^ monocytes to reverse multiorgan fibrosis

**DOI:** 10.1172/jci.insight.151037

**Published:** 2022-03-08

**Authors:** Dan Xu, Swati Bhattacharyya, Wenxia Wang, Igal Ifergan, Ming-Yi Alice Chiang Wong, Daniele Procissi, Anjana Yeldandi, Swarna Bale, Roberta Goncalves Marangoni, Craig Horbinski, Stephen D. Miller, John Varga

**Affiliations:** 1 Department of Microbiology-Immunology and; 2 Northwestern Scleroderma Program, Feinberg School of Medicine, Northwestern University, Chicago, Illinois, USA.; 3 Department of Internal Medicine, University of Michigan, Ann Arbor, Michigan, USA.; 4 Department of Neurological Surgery,; 5 Department of Radiology, and; 6 Department of Pathology, Feinberg School of Medicine, Northwestern University, Chicago, Illinois, USA.

**Keywords:** Autoimmunity, Dermatology, Fibrosis

## Abstract

Systemic sclerosis (SSc) is a chronic, multisystem orphan disease with a highly variable clinical course, high mortality rate, and a poorly understood complex pathogenesis. We have identified an important role for a subpopulation of monocytes and macrophages characterized by surface expression of the scavenger receptor macrophage receptor with collagenous structure (MARCO) in chronic inflammation and fibrosis in SSc and in preclinical disease models. We show that MARCO^+^ monocytes and macrophages accumulate in lesional skin and lung in topographic proximity to activated myofibroblasts in patients with SSc and in the bleomycin-induced mouse model of SSc. Short-term treatment of mice with a potentially novel nanoparticle, poly(lactic-*co*-glycolic) acid (PLG), which is composed of a carboxylated, FDA-approved, biodegradable polymer and modulates activation and trafficking of MARCO^+^ inflammatory monocytes, markedly attenuated bleomycin-induced skin and lung inflammation and fibrosis. Mechanistically, in isolated cells in culture, PLG nanoparticles inhibited TGF-dependent fibrotic responses in vitro. Thus, MARCO^+^ monocytes are potent effector cells of skin and lung fibrosis and can be therapeutically targeted in SSc using PLG nanoparticles.

## Graphical abstract

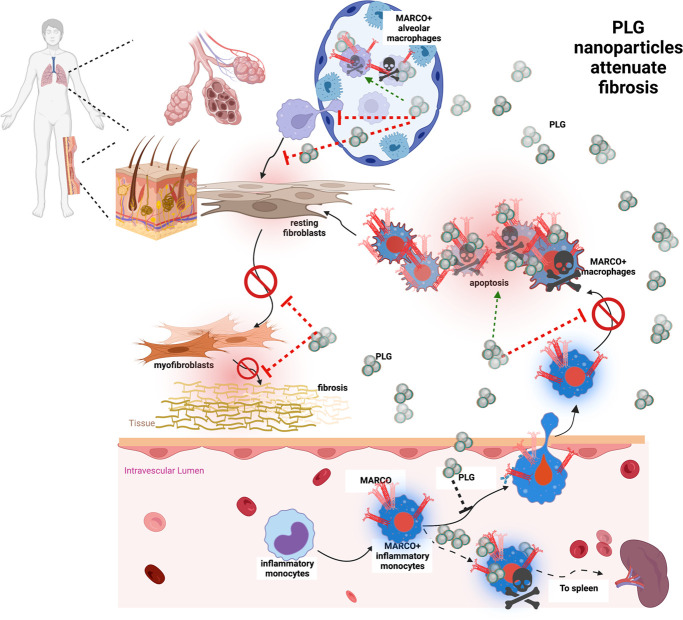

## Introduction

Systemic sclerosis (SSc) is a chronic disease with a poorly understood pathogenesis associated with inflammatory and fibrotic processes in the skin, lungs, and other organs (1–3). Persistent activation of dermal and pulmonary myofibroblasts plays a fundamental role in pathogenesis, but the factors that trigger myofibroblast transformation and prevent their reversion to a state of quiescence with fibrosis resolution remain to be identified (4, 5). GWAS have identified SSc-associated susceptibility loci (6–8), with the majority of genes involved in inflammation and innate immune responses, including TLR signaling and monocyte and macrophage activation and polarization (9, 10). Monocytes and macrophages are a heterogeneous group of bone marrow–derived myeloid cells recruited to sites of tissue injury (11–13). In the skin and lung, they play vital roles in wound healing and response to injury, and their dysregulated activation results in chronic inflammatory and fibrotic pathologies (14, 15).

Upon activation, tissue-specific macrophages and circulating monocytes upregulate expression of macrophage receptor with collagenous structure (MARCO), a type II glycoprotein scavenger receptor that binds to a broad range of anionic ligands (16). In cooperation with TLRs, MARCO plays an important role in innate defenses against microbial pathogens and is also implicated in persistent inflammation (17, 18). Upregulation of MARCO on inflammatory monocytes and macrophages is associated with increased phagocytic capacity, inflammatory cytokine production, and TGF-β secretion (19). To date, to our knowledge, nothing was known regarding the role of MARCO^+^ inflammatory monocytes and macrophages in SSc.

To study the role of MARCO^+^ myeloid cells in the pathogenesis of multiorgan fibrosis in SSc, we developed an innovative therapeutic strategy targeting inflammatory monocytes and activated macrophages by leveraging the drug-like biological properties of carboxylated poly(lactic-*co*-glycolic) acid (PLG) nanoparticles. PLG is an FDA-approved biopolymer used to formulate resorbable sutures. We have shown that negatively charged PLG particles are selectively recognized and bound by inflammatory monocytes and macrophages via MARCO (20). Treatment of mice with PLG induces sequestration of inflammatory monocytes and macrophages in the spleen, where they undergo apoptosis, culminating in reduced immune pathology at peripheral sites of inflammation (20). Acute administration of PLG nanoparticles resulted in reduced inflammatory monocyte accumulation in mouse models of myocardial infarction, acute spinal cord injury, and traumatic brain injury (21–23). Repeated i.v. administration of PLG-derived nanoparticles was well tolerated with no evident adverse effects. The remarkable immunomodulatory properties of the PLG nanoparticles, coupled with their safety and tolerability, recently demonstrated in a celiac disease phase I/IIa clinical trial (24), make them exceptionally appealing candidates for the treatment of inflammation-driven fibrotic diseases.

Here we show that MARCO^+^ monocytes are potent effector cells of skin and lung fibrosis, and that PLG nanoparticles both selectively suppress activated MARCO^+^ monocytes and directly reduce profibrotic cellular signaling. Collectively, our findings define the profibrotic contribution of MARCO^+^ inflammatory monocytes and macrophages in SSc and introduce a well-tolerated and translationally relevant approach to therapy.

## Results

### MARCO^+^ monocytes and macrophages are enriched in the fibrotic skin and lungs of patients with SSc and mice with BLM-induced systemic fibrosis.

**Figure 1:**
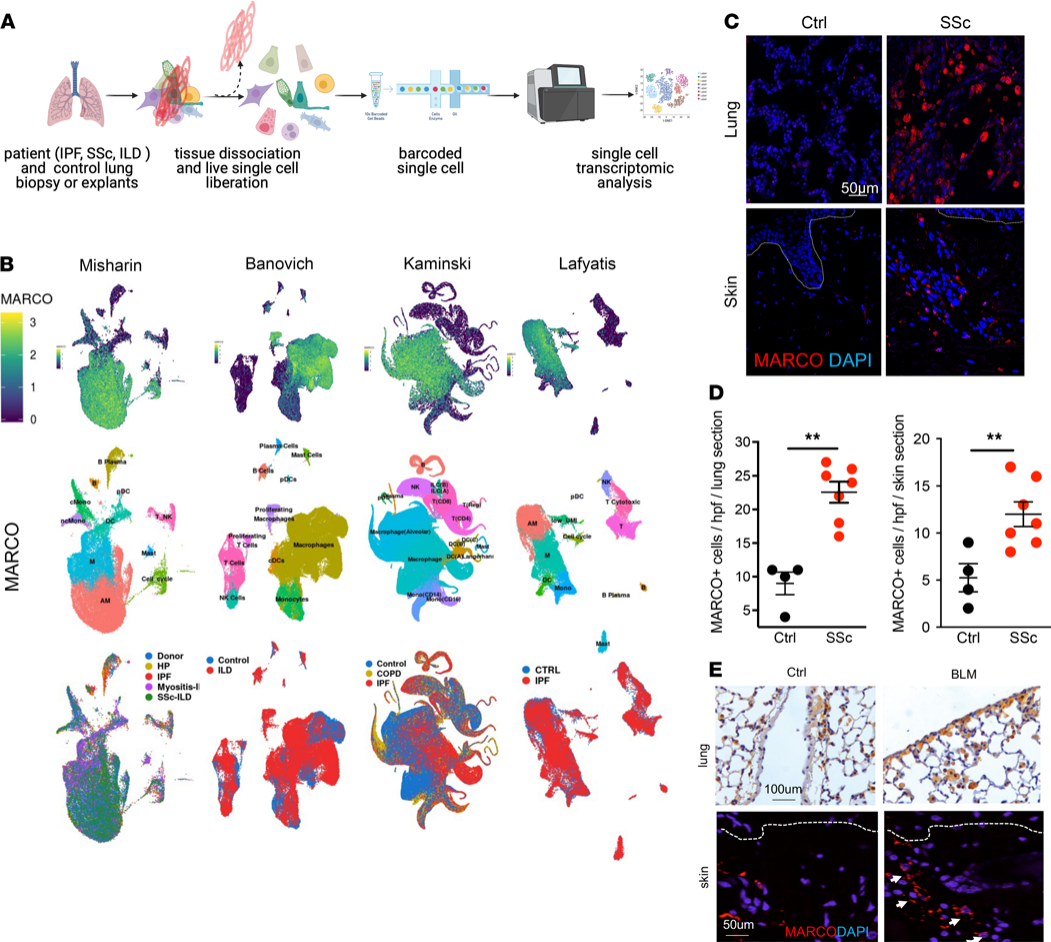
MARCO is upregulated in the lungs and skin of patients with SSc and BLM-injected mice. (**A**) Schematics of experimental design. (**B**) *MARCO* gene expression (top), cell types (middle), and disease status (bottom) of single-cell RNA-Seq data from 4 distinct data sets (Idiopathic Pulmonary Fibrosis Cell Atlas). Each dot represents a single cell. (**C**) Representative confocal immunofluorescent (IF) images of MARCO expression (red) in lung and skin biopsy specimens from patients with SSc (*n* = 7) and control (Ctrl) participants (*n* = 4). Nuclei were identified by DAPI (blue). Scale bar: 50 μm. (**D**) Dot plots of frequency of MARCO^+^ cells (mean ± SEM) determined from 5 high-power fields (hpfs) per section in each biopsy specimen. The *P* values are calculated using the Mann-Whitney *U* test. (**E**) MARCO IHC staining of representative images of lung (brown) and skin tissues (red) from BLM-injected (D21 after injection; *n* = 5 with 3 independent experiments) and Ctrl mice. Dotted lines indicate the dermal-epidermal junction. Scale bars: 100 μm (lung); 50 μm (skin). **P < 0.01. COPD, chronic obstructive pulmonary disease; HP, hypersensitivity pneumonitis; ILD, interstitial lung disease.

In light of recent studies in which researchers have suggested a pathogenic role of persistently activated macrophages and monocytes in SSc (11), we first evaluated the expression of genes related to macrophage and monocyte activation by querying a publicly available single-cell transcriptome data set (25). The Idiopathic Pulmonary Fibrosis Cell Atlas Data Mining Site comprises data generated using single-cell RNA-Seq of lung biopsy specimens from 2 patients with SSc, 59 with idiopathic pulmonary fibrosis (IPF), 18 with obstructive pulmonary disease, 1 with polymyositis, and 1 with chronic hypersensitivity pneumonitis, along with 50 healthy control individuals (Figure 1A) (26–30). Interrogation of single-cell RNA-Seq data from 4 independent groups revealed highly enriched expression of MARCO in circulating and alveolar macrophages, as well as monocytes in both SSc and IPF lungs (Figure 1B and Supplemental Figure 1A; supplemental material available online with this article; https://doi.org/10.1172/jci.insight.151037DS1). Although MARCO was identified over two decades ago, its expression, function, and pathological contribution to SSc, and the therapeutic potential of its targeting, have never been addressed to our knowledge. Therefore, we evaluated the cellular expression of MARCO in SSc lung and skin biopsy specimens. Significant (*P* < 0.01) upregulation of MARCO in SSc lung and skin was noted in comparison with control tissue (Figure, 1 C and D). Immunolabeling of skin biopsy specimens revealed that MARCO expression colocalized with CD68^+^ myeloid cells, which were significantly (*P* < 0.05) enriched in patients with SSc compared with controls (Supplemental Figure 1, B and C). In the skin, MARCO^+^ cells play vital roles in wound healing and response to injury, and their dysregulated activation could result in chronic inflammatory and fibrotic pathologies (31).

To delineate the pathogenic role of MARCO^+^ macrophage and monocytes in SSc, we deployed a bleomycin-induced (BLM-induced) mouse model of the disease (32, 33). Repeated s.c. injections of BLM at 0.5 IU/kg induced pulmonary and dermal fibroses and progressive attrition of dermal white adipose tissue (Figure 1E and Figure 3B). These fibrotic changes in the skin and lungs were accompanied by enrichment of MARCO^+^ cells, with primarily perivascular and interstitial localization.

### Prophylactic PLG nanoparticle treatment attenuates BLM-induced pulmonary and dermal fibroses.

**Figure 2:**
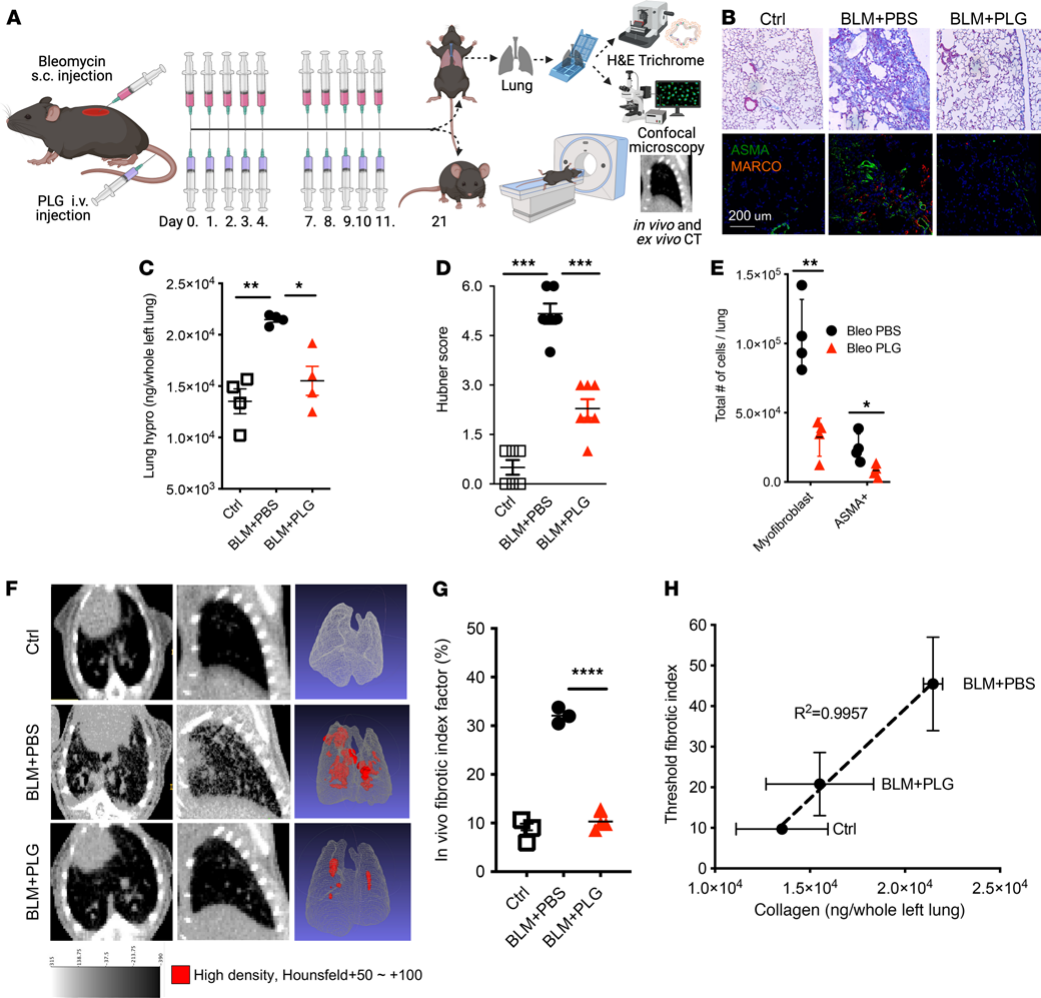
Prophylactic PLG nanoparticle treatment attenuates pulmonary fibrosis in BLM-injected mice. (**A**) Schema of experimental setup and treatment regimen. (**B**) Masson’s trichrome stain (top). Scale bar: 25 μm. Representative images of immunofluorescence staining using Abs to ASMA (green) and MARCO (red) (bottom). Nuclei were identified by DAPI (blue). Scale bar: 200 μm. (**C**) Lung hydroxyproline (hypro) content. *P* values determined via 1-way ANOVA followed by the Šidák’s multiple comparison test. *P < 0.05; **P < 0.01. (**D**) Hubner score. Results are reported as mean ± SD from 5 high-power fields per mouse from at least 5 mice per experimental group. ***P < 0.01. (**E**) Total number of myofibroblasts (live singlets/CD45^–^/PDGFR^+^/CD90^+^) and α-SMA^+^ myofibroblasts per whole left lung per mouse (*n* = 4–5 with 3 independent experiments) measured by flow cytometry. *P < 0.05; **P < 0.01. (**F**) MicroCT of the lungs. Representative cross-sectional (left- and middle-column panels) and 3D images (right-column panels) (**G**) Quantitative lung FI (*n* = 4–5 mice/group with two independent repeats). ****P < 0.0001. (**H**) Correlative comparison between CT-measured FI and half-lung collagen content by hypro assay. Ctrl, control.

To determine the pathogenic contribution of MARCO^+^ myeloid cells to SSc, we exploited the myeloid cell–modulatory properties of PLG nanoparticles (20). Prophylactic treatment with PLG nanoparticles (0.9 mg/mouse i.v.) was initiated concurrently with BLM (Figure 2A). Chronic daily administration of PLG nanoparticles for up to 10 days was well tolerated and no signs of toxicity were observed. As expected, BLM elicited prominent architectural alterations in the lung, including thickened alveolar walls, an influx of leukocytes into the alveoli, the emergence of primarily subpleural localized fibrotic foci, and substantial interstitial collagen deposition (Figure 2, B and C). Markers of pulmonary fibrosis, including collagen content, numbers of α-SMA^+^ myofibroblasts, and Hubner fibrosis score, were all markedly attenuated by PLG treatment (Figure 2, B–E). Importantly, MARCO^+^ cells in topographic proximity to α-SMA^+^ fibroblasts in the lungs were significantly reduced following PLG nanoparticle treatment (Figure 2B).

**Figure 3:**
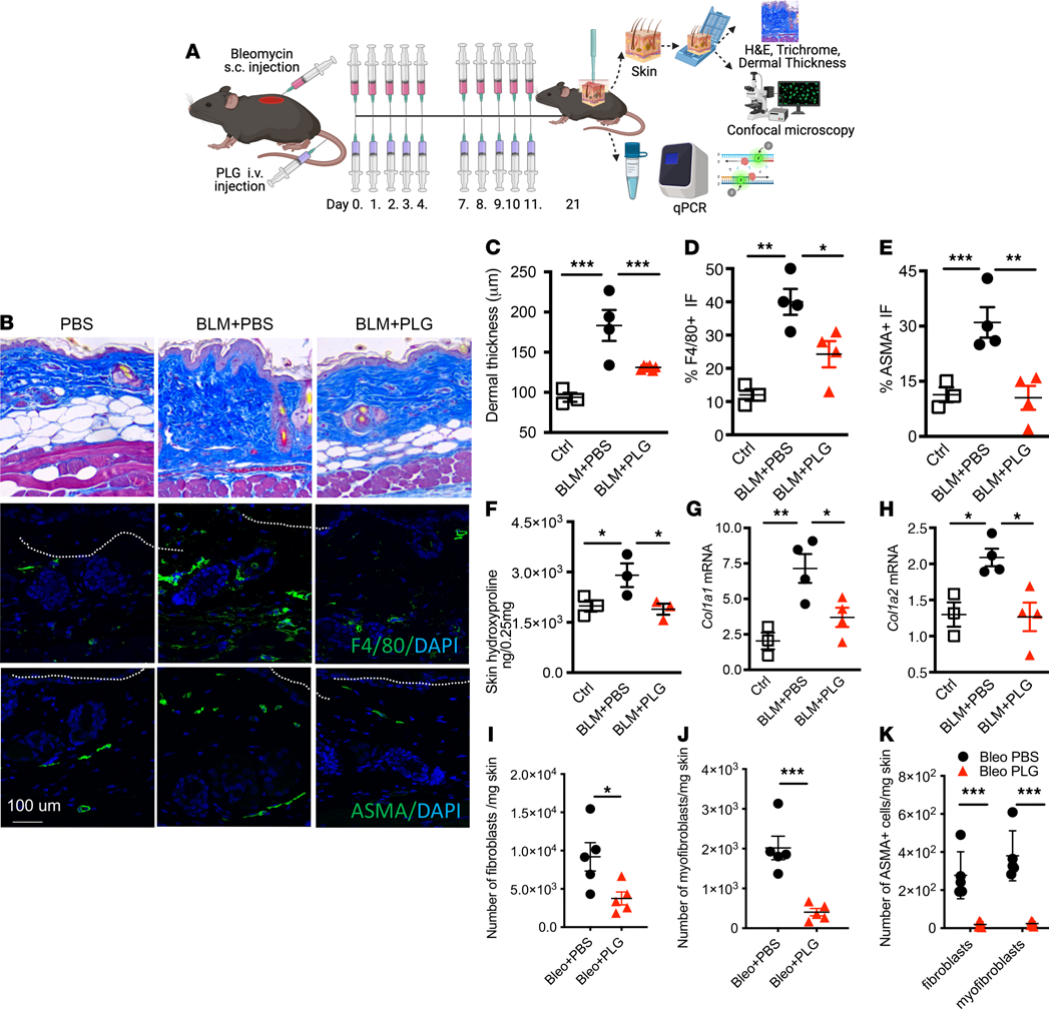
Prophylactic PLG nanoparticle treatment attenuates dermal fibrosis. (**A**) Schematics of experimental design. (**B**) Representative micrographs of Masson’s trichrome stain (top); immunofluorescence (IF) staining for F4/80 (green) with DAPI staining of nuclei (middle); and α-SMA (green) (bottom). (**C**) Dermal thickness (mean ± SEM of 5 determinations per high-powered field (hpf) from 5 mice per group). (**D** and **E**) Dot plots of frequency of F4/80^+^ (**D**) and α-SMA^+^ (**E**) cell populations determined from 5 hpfs in each section. (**F**) Skin hydroxyproline content. (**G** and **H**) Real-time quantitative PCR (qPCR) of *Col1a1* and *Col1a2*. Results, normalized with GAPDH, are reported as mean ± SEM of triplicate determinations from 3 to 4 mice per group. Total number of (**I**) fibroblasts, (**J**) myofibroblasts, (**K**) α-SMA^+^ fibroblasts and α-SMA^+^ myofibroblasts per milligram of skin per mouse (*n* = 4–5 with 3 independent experiments) measured by flow cytometry. *P < 0.05; **P < 0.01; ***P < 0.001 via 1-way ANOVA followed by the Šidák’s multiple comparison test. Ctrl, control.

We next used CT guided by machine learning and artificial intelligence facilitated by histopathology to further evaluate the impact of PLG treatment on lung fibrosis (Figure 2F). An image-based fibrotic index (FI) was generated by subtracting normal lung volume from high-density volume and plotting over total volume as a measurement of fibrotic severity. Mice treated with both PLG nanoparticle and BLM (BLM+PLG) had an FI comparable to that of untreated control mice, suggesting normalized lung architecture, whereas the BLM-injected (BLM+PBS) mice had significantly increased fibrotic lung pathology (Figure 2G). A correlation analysis that compared the lung collagen content (hydroxyproline assay) with the threshold radiologic FI showed that values of PLG-treated mice (BLM+PLG) were similar to that of the control mice (Figure 2H). PLG treatment of mice significantly attenuated the increase of dermal thickness and loss of the dermal white adipose layer induced by s.c. BLM (Figure 3, A–C). The BLM-induced increase in collagen content, accumulation of F4/80^+^ myeloid cells and myofibroblasts, and expression of fibrotic genes within the lesional skin were all significantly reduced in mice treated with PLG nanoparticles (Figure 3, D–H). Most importantly, both the total number and frequency of fibroblasts and myofibroblasts were significantly reduced in PLG nanoparticle–treated mice compared with PBS-treated controls (Figure 3, I–K). Taken together, these findings indicate that prophylactic PLG nanoparticle treatment induced a potent antifibrotic effect.

### Prophylactic PLG nanoparticle treatment restricts the accumulation of activated immune cells in fibrotic tissues.

**Figure 4:**
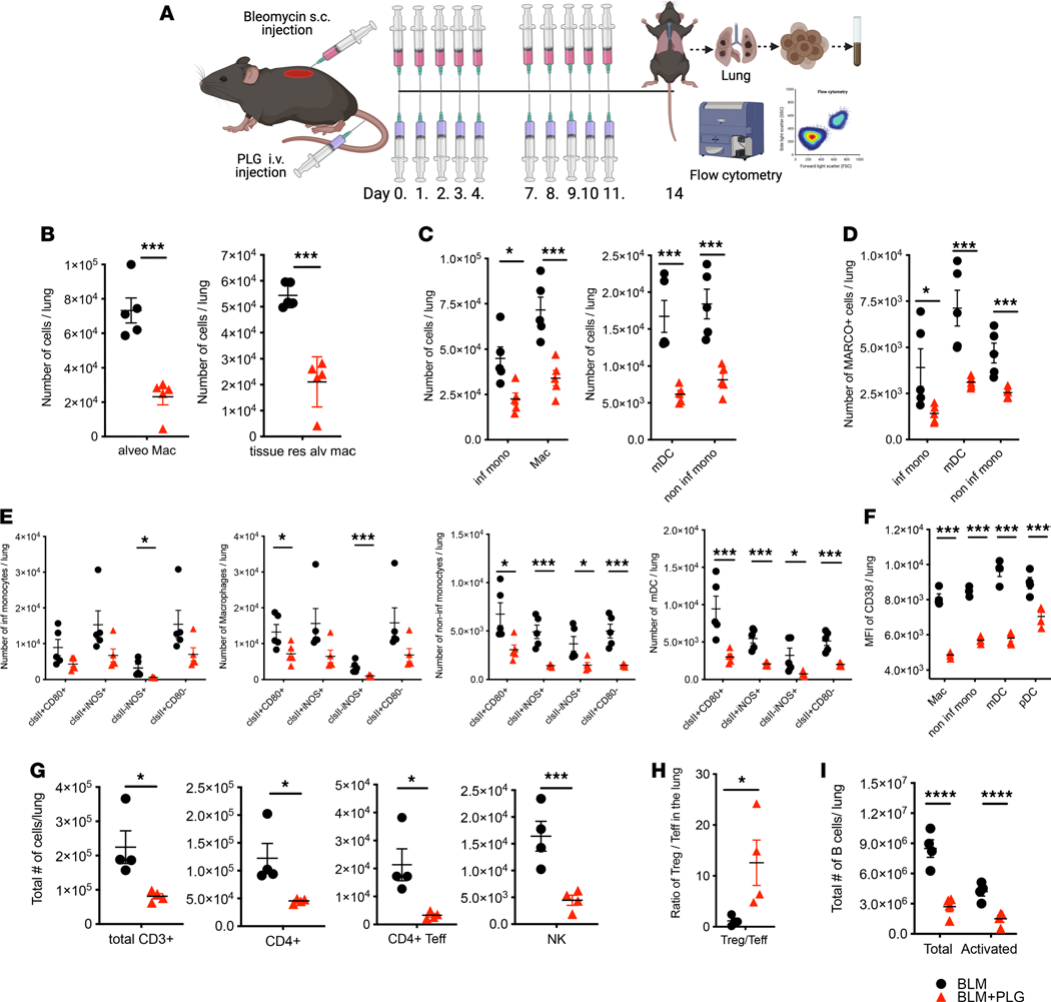
Prophylactic PLG nanoparticle treatment reduces immune cell infiltrates and tissue-resident macrophages (macs) in the lungs of BLM-treated mice. (**A**) Schema of experimental setup and treatment regimen. (**B**) Number of total alveolar (alv) macs and tissue-resident (res) alv macs per lung per mouse. (**C**) Total number of myeloid lung infiltrates, including inflammatory monocytes (inf monos), macs, myeloid DCs (mDCs), and noninflammatory monocytes (noninf mono). (**D**) MARCO^+^ myeloid immune infiltrates. (**E**) Quantitation of differentially activated myeloid cell subsets expressing MHC II (clsII), CD80, and iNOS. (**F**) Level of cell surface expression of CD38 protein measured by MFI in various myeloid immune infiltrates in the lung, including macs, noninf monos, mDCs, and plasmacytoid DCs (pDCs) (**G**) Total number of lymphoid immune infiltrates, including CD3^+^ T cells (CD3^+^), CD4^+^ T cells (CD4^+^), CD4^+^ Teffs, and NK cells. (**H**) Ratio of the number of Tregs to Teffs. (**I**) Total number of B cells in the lung. All graphs were generated by flow cytometric analysis (*n* = 4–5 mice per group with 3 independent experiments), with data reported as mean ± SEM. *P < 0.05; **P < 0.01; ***P < 0.001; ****P < 0.0001 via 1-way ANOVA followed by the Šidák’s multiple comparison test.

Cellular mediators that connect early inflammation with fibrosis represent potential therapeutic targets in SSc (34–39). We thus evaluated the antiinflammatory effects of PLG nanoparticle treatment on immune cell phenotypes, differentiation, activation, and function. We focused on d14 post-BLM injection (Figure 4A), a time point chosen on the basis of time-course studies showing that immune cell infiltration and activation in the lung and skin were comparable with that seen on d21 (Supplemental Figure 2).

Treatment with PLG nanoparticles significantly reduced the numbers of CD45^+^ leukocytes in the lungs of BLM-treated mice while preventing dramatic cell loss in the spleens (Supplemental Figure 3). PLG treatment also significantly reduced the numbers of tissue-resident alveolar macrophages, peripheral monocyte–derived alveolar macrophages, inflammatory monocytes, noninflammatory monocytes, and conventional DCs (Figure 4, B–D). Notably, the numbers of each of the myeloid subsets expressing activation markers (MHC II, iNOS, and CD80) were also significantly attenuated by PLG treatment (Figure 4E). Interestingly, PLG treatment significantly reduced IL-1 production while increasing the frequency of IL-12–secreting antigen-presenting cells (APCs) in the spleen, suggesting a phenotypic skewing toward M1 for APCs and Th1 for CD4^+^ T cells (Supplemental Figure 4). It is noteworthy that there was a significant loss of MARCO^+^ myeloid cells, consistent with histologic analysis and corroborating the clinical data (Figure 4D). Decline of antiaging histone deacetylases SIRT1 and SIRT3 has been detected in patients with SSc and associated with fibrosis induction. Treatments that augment SIRT1 and SIRT3 activity via inhibition of NAD hydrolase (CD38) activity represent a promising antifibrotic strategy. PLG nanoparticles markedly downregulated CD38 expression in the lung-infiltrating myeloid cells, suggesting PLG’s potential as an antifibrotic strategy through regulation of cellular senescence and metabolism (Figure 4F).

In addition to the myeloid compartment, we also interrogated the lymphocyte subsets, most notably Tregs, effector CD4^+^ T cells, and NK cells. We found that PLG nanoparticle treatment caused a global reduction of lymphocytes, including total CD3^+^ T cells, total and activated effector CD4^+^ T cells (Teffs), and NK cells (Figure 4G). Remarkably, the Treg to Teff ratio was significantly augmented after PLG treatment, suggesting a potential protective role of Tregs in early fibrogenesis (Figure 4H). PLG treatment also mitigated the BLM-induced increase in B cell numbers and activation state (Figure 4I). Additionally, alteration in cellular energy metabolism and senescence has been suggested in SSc and animal models of SSc (40–42).

**Figure 5:**
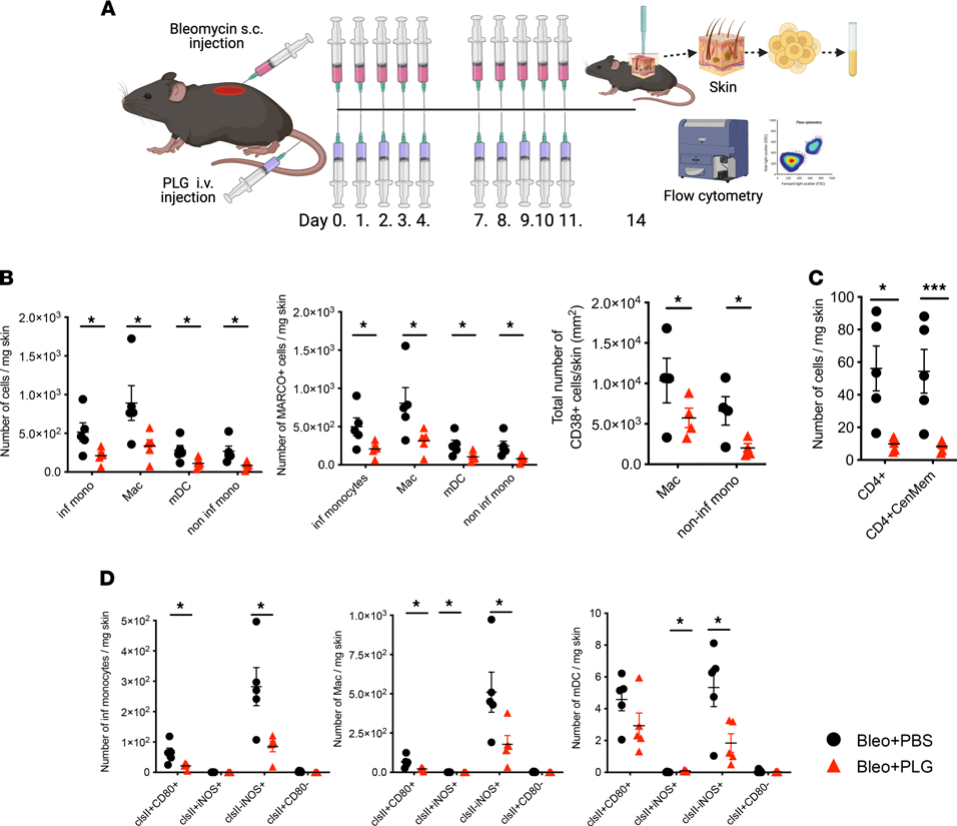
Prophylactic PLG nanoparticle treatment reduces immune cell infiltrates in the skin of BLM-treated mice. (**A**) Schema of experimental setup and treatment regimen. (**B**) Total number of myeloid subset infiltrates (left panel), including inflammatory monocytes (inf monos), macrophages (macs), myeloid DCs (mDCs), and noninflammatory monocytes (noninf monos). Middle panel shows quantitation of MARCO^+^ myeloid immune infiltrates. Right panel shows CD38^+^ macs and noninf monos. (**C**) Total number of CD4^+^ T cell infiltrates, including CD4^+^ T cells (CD4^+^) and central memory (CenMem) CD4^+^ T cells (CD4^+^CenMem). (**D**) Quantitation of differentially activated myeloid cell subsets using expression of MHC II (clsII), CD80, and iNOS. All data were generated by flow cytometric analysis (*n* = 4–5 mice/group with 3 independent experiments), with data reported as mean ± SEM. *P < 0.05; ***P < 0.001 via 1-way ANOVA followed by the Šidák’s multiple comparison test.

Prophylactic PLG administration in BLM-treated mice had comparable effects in the skin, with marked reduction in the numbers and activation state of myeloid cells (Figure 5, A, B, and D). Similar to the CD4^+^ Teff reduction seen in the lung, BLM-induced increase in central memory CD4^+^ cells in the skin were also reduced (Figure 5C). PLG treatment also resulted in a significant decrease in the numbers of α-SMA^+^ fibroblasts and myofibroblasts in the skin (Figure 3, E and K). This illustrates the efficiency of the PLG nanoparticles in modulating memory T cells, which are mostly resistant to immune regulatory therapies and whose function and numbers are difficult to alter.

### Reversal of established fibrosis by therapeutic PLG nanoparticle treatment.

**Figure 6:**
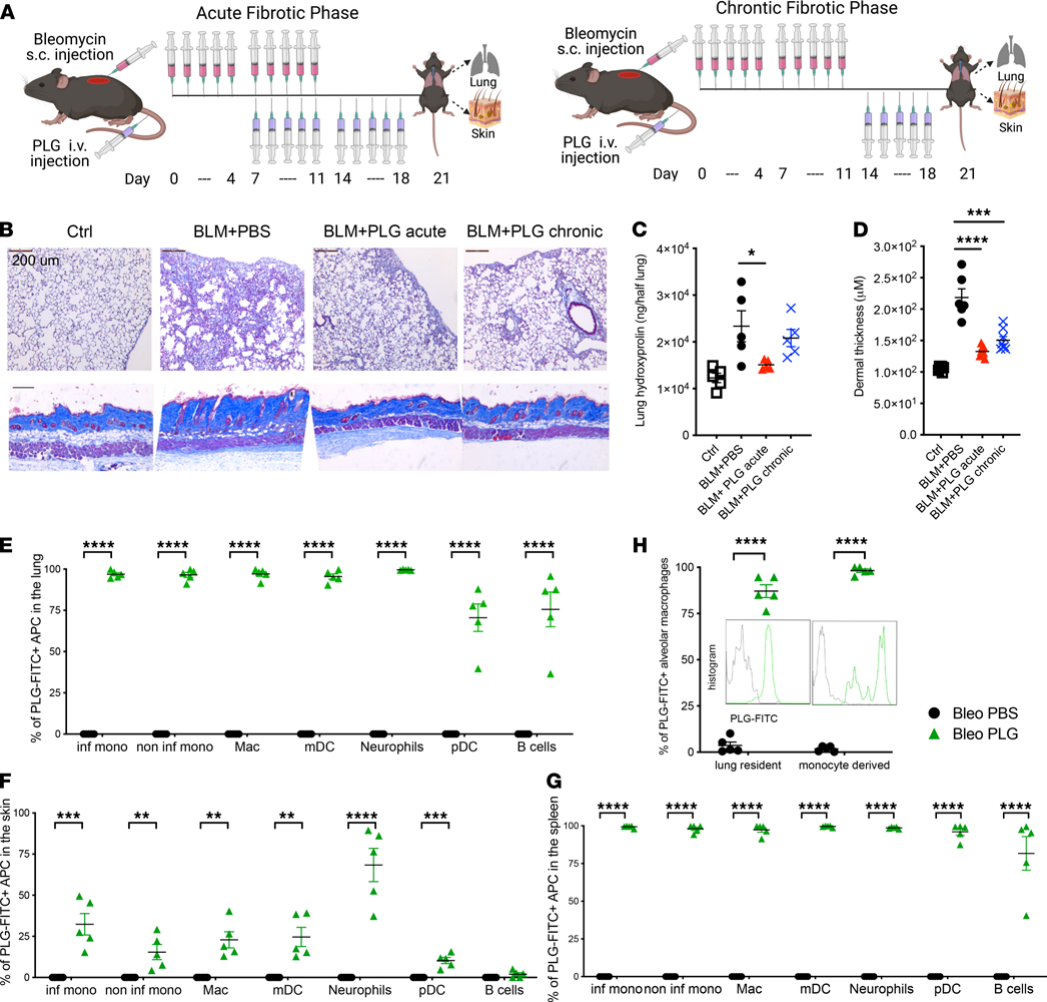
Therapeutic PLG nanoparticle treatment during the acute and chronic fibrotic phases attenuates pulmonary and dermal fibrosis. (**A**) Schema of experimental setup and treatment regimens for acute and chronic phases of disease. (**B**) Representative images of Masson’s trichrome stain of the lung (top panels) and skin (bottom panels). Scale bar: 200 μm. (**C**) Lung hydroxyproline content and (**D**) dermal thickness (reported as mean ± SEM of 5 determinations per high-powered field from 5 mice per group). Percentage of PLG-FITC^+^ APCs in the lung (**E**), skin (**F**), and spleen (**G**) of control (Ctrl) and PLG-FITC^+^–injected mice. (**H**) Frequency and MFI of PLG-FITC^+^ alveolar macrophages (macs) in the lung. *P < 0.05; ***P < 0.001; ****P < 0.0001 via 1-way ANOVA followed by the Šidák’s multiple comparison test. inf mono, inflammatory monocyte; mDC, myeloid DC; noninf mono, noninflammatory monocyte; pDC, plasmacytoid DC.

To assess the effect of PLG on preexisting fibrosis, treatment was initiated either during the acute (d7) or the chronic phase (d14) of BLM treatment, when dermal and pulmonary fibroses are already detectable (Figure 6A). PLG treatment started at either time point significantly attenuated BLM-induced increase in collagen deposition in the lung and skin (Figure 6, B and C), mitigated the distortion of pulmonary architecture as well as infiltration of leukocytes, and attenuated dermal thickening (Figure 6, B and D).

To determine whether the therapeutic effects are derived in part from directly targeting splenic myeloid cells or MARCO^+^ cells in the fibrotic lung and skin, we performed tracing studies using FITC-labeled PLG (FITC-PLG) nanoparticles to understand their tissue and cellular distribution. Mice were injected with 10 doses of BLM s.c. from d0 to d11 and 5 doses of FITC-PLG nanoparticles i.v. from d7 to d11. An additional dose of FITC-PLG nanoparticles was given i.v. 1 hour before tissue harvest on d14 to ensure the detection of intracellular fluorescent signal, which diminishes 3 hours after each dosing of FITC-PLG nanoparticles, due to the biodegradable nature of the PLG nanoparticle (our unpublished observation). FITC-PLG^+^ APCs were readily detected by flow cytometry in the fibrotic lung, skin, and spleen (Figure 6, E–G). Remarkably, FITC–PLG nanoparticle uptake was also detected in alveolar macrophages, both lung resident and monocyte derived, suggesting a direct effect of PLG nanoparticles in tissue-resident myeloid cells (Figure 6H).

### PLG nanoparticles directly modulate myofibroblast activation via pSTAT.

**Figure 7:**
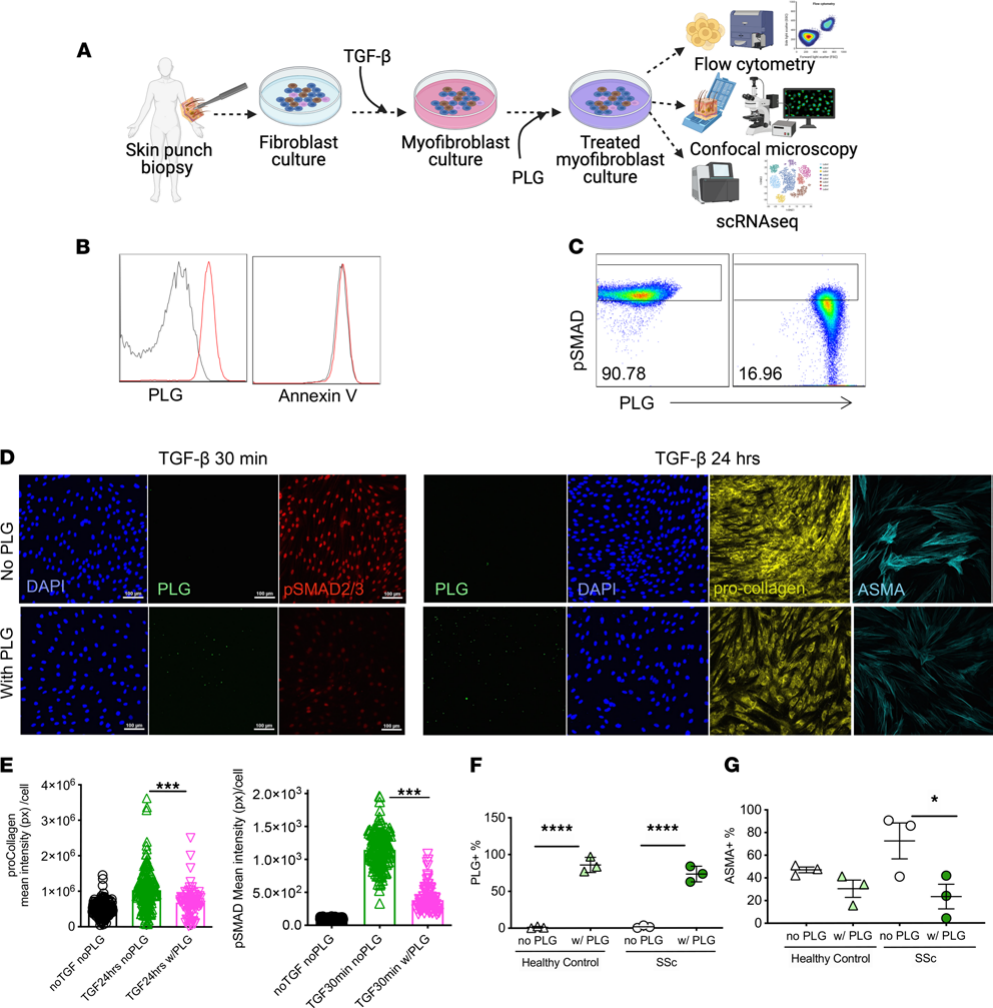
PLG nanoparticles mitigate collagen production by myofibroblasts via reduction of pSMAD2/3. (**A**) Schematics of experimental design. (**B**) PLG uptake and apoptosis evaluation (annexin V^+^) by cultured fibroblasts isolated from foreskin of healthy individuals and measured by flow cytometry. Representative histogram of PLG^+^ and annexin V^+^ cells (no PLG control: gray; with PLG: red; *n* = 3). (**C**) Levels of phosphorylated SMAD2/3 (pSMAD2/3) in TGF-β–treated fibroblast cultures in the presence and absence of PLG nanoparticles (10 g/mL). (**D**) Representative confocal micrographs of intracellular PLG content (green), pSMAD (red), procollagen (yellow), α-SMA (cyan), and DAPI (blue) in PLG-treated and control fibroblasts. (**E**) Quantified procollagen and pSMAD2/3 levels by confocal microscopy. *n* > 850 cells/sample. (**F**) Frequency of PLG^+^ fibroblasts from skin biopsy specimens from healthy control individuals and patients with SSc. *n* = 3/group. (**G**) Frequency of α-SMA after PLG nanoparticle treatment in fibroblasts from skin biopsy specimens from healthy control individuals and patients with SSc. *P < 0.05; ***P < 0.001; and ****P < 0.0001 via 1-way ANOVA followed by the Šidák’s multiple comparison test. px, pixel.

Myofibroblasts play a central role in fibrosis initiation and progression in SSc and other fibrotic diseases (43). To investigate the potential of PLG nanoparticles to directly affect myofibroblast differentiation and function, we used cultures of foreskin as well as healthy and SSc adult skin fibroblasts (Figure 7A). Confluent fibroblasts in vitro were incubated with PLG (10 g/mL) ex vivo for 1 hour. The cellular uptake of PLG nanoparticles was comparable in foreskin fibroblasts and healthy control or SSc skin fibroblasts without inducing apoptosis or necrosis (Figure 7B).

Reprogramming of mesenchymal progenitor cells to fibrotic myofibroblasts is triggered by TGF-β (44). To investigate the mechanisms underlying the observed ant-fibrotic effects, we thus focused on canonical signaling. Incubation with TGF-β induced rapid Smad2/3 phosphorylation and nuclear localization in normal fibroblasts (Figure 7, C–E). PLG nanoparticles, even when added to the cultures after exposure to TGF-β for 24 hours, induced marked downregulation of fibrotic cellular responses (Figure 7, D and E). Importantly, PLG nanoparticle treatment significantly reduced α-SMA expression in SSc, but not in healthy control fibroblasts, despite comparable nanoparticle uptake (Figure 7, F and G).

**Figure 8:**
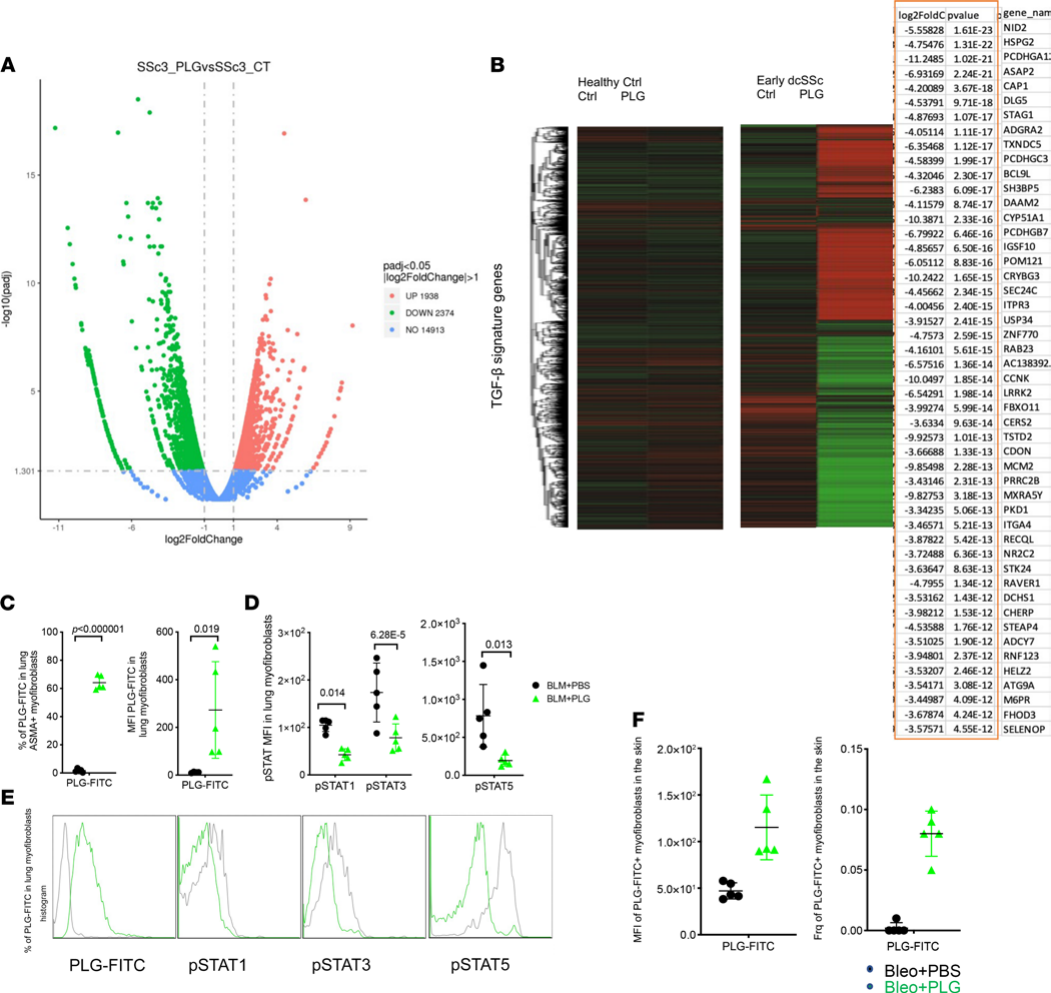
PLG nanoparticles mitigate collagen production by PLG-FITC^+^ myofibroblasts via reduction of pSTAT. Confluent human skin fibroblasts isolated from skin biopsy specimens from healthy control (Ctrl) individuals and patients with SSc were treated with PLG nanoparticles or PBS as Ctrl for 1 hour before bulk mRNA was isolated and subjected to genome-wide transcriptome analysis. (**A**) The volcano plot shows differentially up- or downregulated genes in an unsupervised cluster analysis of TGF-β pathway signature genes before and after PLG nanoparticle treatment (**B**) Heatmap demonstrates altered expression of genes. Red indicates higher and green indicates lower levels of gene expression. The top 50 downregulated TGF-β signature genes are listed in the table. (**C**) The percentage and MFI of PLG-FITC^+^ myofibroblasts in the lung. (**D** and **E**) The MFI and representative flow plots of pSTAT1, pSTAT3, and pSTAT5 expression in the lung fibroblasts isolated from Ctrl and PLG-FITC^+^–treated mice. (**F**) The frequency and MFI of PLG-FITC^+^ myofibroblasts in the skin of Ctrl and PLG-FITC–treated mice. *P < 0.05; ***P < 0.001; ****P < 0.0001 via 1-way ANOVA followed by the Šidák’s multiple comparison test. dcSSc, diffuse cutaneous systemic sclerosis; padj, adjusted *P* value.

To further characterize the impact of PLG on fibroblast responses, an unbiased genome-wide RNA-Seq analysis was performed. PLG treatment induced marked alteration in the transcriptome profile of SSc fibroblasts, as shown by the volcano plot and the heatmap in Figure 8, A and B. TGF-β family genes were substantially downregulated in PLG-treated SSc fibroblasts, whereas healthy control fibroblasts showed more modest transcriptome alterations in response to PLG (Figure 8, A and B).

The therapeutic effect of PLG nanoparticles clearly points toward a fibrotic reduction and reversal mechanism independent of myeloid targeting. To investigate the potential mechanism underlying directing of myofibroblasts in the absence of MARCO expression (<5% fibroblasts express MARCO; our unpublished observation), we used FITC-PLG nanoparticles to track nanoparticle uptake by fibroblasts and myofibroblasts. Tissue-resident fibroblasts and, more remarkably, myofibroblasts (α-SMA–positive [ASMA^+^] fibroblasts) engulfed FITC-PLG nanoparticles in the fibrotic lung and skin after therapeutic PLG treatment (Figure 8, C and F). This resulted in a significant downregulation of pSTAT 1, 3, and 5 (Figure 8, D and E).

These data support the direct therapeutic effect of PLG nanoparticles on fibroblasts during the chronic phase of SSc when immune infiltration has subsided. Together, these findings demonstrate a potentially novel therapeutic function of PLG nanoparticles as potent inhibitors of inducible and constitutive Smad-dependent and pSTAT-relevant fibrotic responses in normal and SSc skin fibroblasts.

## Discussion

We describe a previously undefined critical pathogenic role for MARCO^+^ myeloid cells in skin and lung fibrosis in SSc. Moreover, targeted reduction of cutaneous and pulmonary infiltration of MARCO^+^ inflammatory monocytes and macrophages using an innovative PLG nanoparticle strategy effectively prevented and, more importantly, reversed skin and lung pathology in a BLM-induced mouse model of fibrosis. PLG nanoparticles displayed an outstanding safety profile in mice with severe multiorgan fibrosis and significant weight loss. In addition to modulation of MARCO^+^ cells, the biodegradable PLG nanoparticles simultaneously downregulated multiple fibrosis-inducing pathways through attenuation of myofibroblast transformation, mitigation of fibroblast activation, induction of Th1-skewed CD4^+^ T cell polarization, boosting of Tregs, depletion of tissue-infiltrating B cells, and rescue of multilineage cell senescence.

Blood-borne immune cells are commonly found within early-stage SSc lesions and are thought to secrete soluble mediators to initiate fibrotic responses (11). It is not known whether regulation of these cells later in the fibrotic process could substantially ameliorate the disease. Here, we show that prophylactic PLG nanoparticle treatment significantly inhibited the accumulation of various myeloid cell subsets in the lung and skin of BLM-injected mice. Interestingly, treatment initiated during the chronic fibrotic phase was equally effective as the prophylactic PLG treatment, suggesting the continuous contribution of myeloid cells in chronic pathogenesis of SSc and the potential of targeting this population for therapeutic purposes, even during the later stages of fibrosis. Blood-borne monocytes and bone marrow–derived macrophages have long been implicated in the pathogenesis of SSc, but the potential contribution of long-lived, tissue-resident macrophages to fibrosis is just beginning to be appreciated (45). PLG nanoparticle treatment significantly reduced the accumulation of both circulating monocytes and tissue-resident macrophages in the lung. Consistent with our results, several groups have reported that deleting monocyte-derived alveolar macrophages within an injured lung can lessen the severity of fibrosis (45–47).

PLG nanoparticles constitute a selective treatment that specifically reduces inflammatory monocytes and alveolar macrophages in the chronically inflamed lung without requiring a systemic depletion protocol. Although the detailed mechanism of lung protection from fibrotic injury as a result of reduced cellularity of inflammatory monocytes and alveolar macrophages merits further investigation, the therapeutic effect of PLG nanoparticles is not just the result of targeting inflammatory monocytes and other myeloid cells in the spleen and the circulation alone but also the ability of particles to gain access to tissue-resident APCs under inflammatory conditions (Figure 6). Importantly, PLG nanoparticle–induced diminution resulted in a significantly improved pulmonary histology in treated mice compared with controls.

MARCO appears to be a potentially novel therapeutic marker that can be targeted to halt the progression of pulmonary fibrosis; it has been shown to promote macrophage polarization to a profibrotic M2 phenotype and precipitate an exaggerated fibrotic response to lung injury in a model of asbestosis (19). PLG nanoparticle treatment not only significantly reduced MARCO^+^ myeloid cells, including macrophages, inflammatory monocytes, and conventional DCs, but, more importantly, reduced macrophage activation as indicated by reduced expression of MHC class II, costimulatory molecules, and iNOS. Mounting evidence suggests that altered cellular senescence and aberrant metabolic reprogramming contribute to SSc pathogenesis. Senescent cells accumulate in connective tissues to enhance extracellular matrix production and facilitate production of proinflammatory and profibrotic mediators, including IL-6 and TGF-β (48). In patients with SSc and in genetically modified animals, reduced activity of antiaging histone deacetylases SIRT1 and SIRT3 is directly correlated with severe fibrosis (49–51). The decline in SIRT1 and SIRT3 activity is linked to NAD depletion as a result of enhanced expression of the ectoenzyme NAD hydrolase, CD38. Treatments that boost NAD levels or inhibit NAD hydrolase activity have been shown in preclinical models to be efficacious in the control of fibrosis development or progression (52). Interestingly, PLG nanoparticles robustly reduced CD38 expression in immune cells and nonhematopoietic stromal cells in the skin and lung of BLM-induced mice, indicative of the antifibrotic potential of this therapy.

Macrophage polarization is tightly regulated by various inflammatory factors, one of which is the differential signaling by Th1 and Th2 cytokines (53, 54). PLG nanoparticle treatment reduced splenic levels of IL-6 while enhancing IL-12 production, a Th1-inducing cytokine. Longitudinal analysis of serum cytokines in patients with SSc has revealed that elevation of the Th1-skewing cytokine IL-12 is associated with spontaneous regression of skin sclerosis, whereas low IL-12 levels correlated with increased mortality (55). PLG nanoparticles effectively pushed CD4^+^ T cells toward a Th1-skewed phenotype and inhibited Th2 development and cytokine production, a condition critical for suppressing M2 macrophage induction, contributing to amelioration and reversal of fibrosis. PLG nanoparticles also significantly boosted the numbers of Tregs. During a steady state, skin and lung are among the nonlymphoid organs that harbor the most Tregs. According to findings of several studies, Tregs from patients with SSc may have impaired suppressive capacity for Teffs and correspondingly produce reduced levels of inhibitory cytokines (56, 57). Augmenting Tregs led to a decrease in skin fibrosis, which closely resembles the pathophysiology of SSc, in patients with chronic graft-versus-host disease. The Treg to Teff ratio was significantly elevated in the lungs of PLG nanoparticle–treated mice compared with controls. An enhanced Treg to Teff ratio is often used as a benchmark for effective T cell–mediated immunoregulatory therapy. These findings suggest that PLG nanoparticle treatment regulated both the Treg and Teff compartments, inhibiting the development of and even reversing skin fibrosis in mice.

Another arm of adaptive immunity that contributes to the pathogenesis of SSc is Ab-producing B cells (58–60). PLG nanoparticle treatment led to a significant reduction in total and activated B cells in the lungs and skin of BLM-injected mice. The numbers of activated B cells that expressed MHC class II and costimulatory molecules were also significantly reduced. Similarly, B cell depletion and blockade therapies targeting CD20, CD19, or B cell–activating factor of the TNF family have shown promising results in clinical trials resulting in improved SSc outcomes (61–63). Although PLG nanoparticles are effective at systemically reducing the numbers of total and activated B cells, further investigation is required to determine whether memory plasma cells in the bone marrow, which are the main source of long-term Ab production, can be readily modulated by nanoparticle treatment.

Activated myofibroblasts are the principal drivers of extracellular matrix remodeling. Therapies that control their function and survival are of great relevance to development of drugs for SSc treatment. We found that PLG nanoparticle treatment directly regulated TGF-β via pSMAD and pSTAT, canonical signaling pathways in fibrogenesis. PLG nanoparticles can be internalized not only by immune cells but also stromal cells, such as fibroblasts (Figures 7 and 8). However, the phenotypic and functional outcomes after nanoparticle uptake by immune and nonimmune cells are disparate. Nonimmune cells do not undergo apoptosis (our personal observations). Instead, they downregulate pSTAT1, 3, and 5 (Figure 8). Increased pSTAT3 has been reported in SSc skin biopsy specimens and mouse models (64). Inhibition of pSTAT3 using a STAT inhibitor led to attenuated skin fibrosis, reduced profibrotic gene expression, and ameliorated collagen deposition. In addition to the pSTAT pathway, PLG nanoparticles also altered the phosphorylation status of SMAD2 and SMAD3 following TGF-β treatment to explanted primary fibroblast cultures generated from skin biopsy specimens from healthy individuals and patients with SSc for over 25 hours, a time point at which the TGF-β pathway has been activated for effective driving of myofibroblast differentiation. Inhibition of SMAD phosphorylation and reversal of myofibroblast activation are unprecedented in antifibrotic drug development. These outcomes are a result of direct nanoparticle uptake by fibroblasts and translocation of PLG nanoparticles to the lysosome (Figure 8, C–F, and our personal observations). It is important that PLG nanoparticles robustly downregulated α-SMA production in fibroblasts isolated from patients with SSc, whereas those from healthy control individuals were only mildly affected, suggesting a disease-specific modification instead of interference in normal wound healing. This finding further strengthens the safety profile of PLG nanoparticle administration.

Our FITC-PLG nanoparticle tracking data suggest the effect of PLG on fibrosis is biphasic. During the early phase of fibrosis, in which immune response is the predominant driving force of fibrotic progression, PLG targets inflammatory monocytes and macrophages. If treatment was initiated during the chronic phase, when immune infiltration has subsided and myofibroblasts are the main driver of fibrosis, PLG nanoparticles directly targeted fibroblasts, and even myofibroblasts, by downregulating the STAT pathways to modulate and reverse fibrosis.

This study provides potentially novel evidence that MARCO^+^ monocytes and macrophages are cellular subsets critical for fibrosis induction and progression in SSc. Importantly, we describe a biodegradable, nanoparticle-based therapy with an outstanding safety profile that simultaneously modifies immune cells and fibroblasts. This therapeutic strategy thus operates via multiple pathways for fibrosis management, including attenuation of fibroblast activation via downregulation of pSMAD, boosting of immunoregulatory Tregs, reduction of Th2 cytokines while inducing a Th1-skewed CD4^+^ T cell phenotype, depletion of Ab-producing B cells, and reduction of cell senescence–related CD38 expression in stromal and immune compartments. To our knowledge, this is the first report of a novel therapeutic approach for treatment of SSc using a nanomaterial strategy that targets multiple cell lineages contributing to fibrosis formation and progression.

## Methods

### BLM-induced mouse model of systemic fibrosis.

C57Bl/6 mice from The Jackson Laboratory were used for all the experiments. For in vivo experiments, mice were randomized to receive control (PBS+PBS), BLM (BLM+PBS), or BLM in combination PLG (BLM+PLG; 0.9 mg/mouse i.v.; Phosphorex). Mice were fed a standard chow diet and given daily s.c. injections of PBS or BLM (0.5 IU/kg; Teva Pharmaceuticals, North Wales, PA) for 10 days. Mice were sacrificed on day 14 for flow cytometry analysis and on day 21 for histologic evaluation of lesional skin and lungs. Tissue collagen content was determined by hydroxyproline assays, as previously described (65).

### Cell cultures.

Primary cultures of human dermal fibroblasts were established from explanted neonatal foreskin or adult forearm skin biopsy specimens from patients with SSc or age-matched healthy control individuals. Early-passage fibroblasts (from fewer than 3 passages) were grown in monolayers in 6-well plastic dishes or 100-mm plates (Corning Inc.) and studied at early confluence. Cultures of human fibroblasts were maintained in DMEM supplemented with 10% FBS (Thermo Fisher Scientific), 1% vitamin solutions, 2 mM l-glutamine, and 120 units/mL penicillin and streptomycin (Lonza).

To test fibrotic response, confluent fibroblasts were incubated in serum-free medium containing 10 ng/mL TGF-β (Peprotech; catalog 100-21) and 0.1% BSA (MilliporeSigma). PLG nanoparticles were added either 16 hours after TGF-β for the collagen I confocal experiment or simultaneously with TGF-β for the pSmad 2/3 assay.

### RNA isolation and quantitative PCR analysis.

Total RNA from cultured human and mouse fibroblasts, macrophages, or mouse tissues was isolated by Quick RNA MiniPrep Kit (Zimo Research; catalog 11-328) or by RNeasy Fibrous tissue Mini Kits (Qiagen; catalog 74704). Reverse transcription of RNA to cDNA was performed using Supermix (cDNA Synthesis Supermix; Quanta Biosciences), as previously described (65). Amplification products (50 ng) were amplified using SYBR Green PCR Master Mix or TaqMan gene expression assay (Applied Biosytems) on an Applied Biosystems 7500 Prism Sequence Detection System. Gene expression was normalized to internal GAPDH, and the amount of change was calculated. Primers were synthesized by Integrated DNA Technologies and the sequences were as follows: m*Col1A1* RT-qPCR forward 5′-AGCCGCAAAGAGTCTACATG-3′; m*Col1A1* reverse-transcriptase quantitative PCR (RT-qPCR) reverse: 5′-CTTAGGCCATTGTGTATGCAG-3′; m*Col1A2* RT-qPCR forward 5′-CCGTGCTTCTCAGAACATCA-3′; m*Col1A2* RT-qPCR reverse 5′-CTTGCCCCATTCATTTGTCT-3′; mGAPDH RT-qPCR forward 5′-ATCTTCTTGTGCAGTGCCAGC-3′; and m*GAPDH* RT-qPCR reverse 5′-GTTGATGGCAACAATCTCCAC-3′.

### Immunofluorescence confocal microscopy.

Foreskin fibroblasts were seeded on glass coverslips and studied by immunofluorescence as previously described (66). Fibroblasts at 80% confluency were fixed in 3.7% paraformaldehyde, permeabilized, and incubated with Abs to α-SMA (MilliporeSigma) or phospho-Smad2 (Cell Signaling Technology Inc.; catalog 3018) at a 1:100 dilution, followed by Alexa Fluor–labeled secondary Abs (Invitrogen). Nuclei were counterstained with DAPI. Subcellular distribution of immunofluorescence was evaluated under an immunofluorescence microscope, a Zeiss UV Meta 510 confocal microscope, or a Nikon C2 or A1Si confocal microscope and quantitated using ImageJ (NIH).

### Histology and IHC.

We analyzed 4 μm sections of FFPE skin biopsy samples from patients with SSc, healthy individuals, or from mouse skin tissue and lungs by IHC, as previously described (65). Slides were incubated with anti-mouse CD38 (Abcam; catalog ab230153), anti-mouse or human ASMA (MilliporeSigma; catalog A2547), F4/80 (Cell Signaling; catalog 70076T), anti-human MARCO (Abcam; catalog ab231046), anti-mouse MARCO (Abcam; catalog ab256822), and mouse CD68 (Abcam; catalog ab125212) Abs or isotype-matched control IgG (eBioscience), followed by HRP-conjugated secondary Ab, which was visualized with diaminobenzidine substrate and counterstained with hematoxylin. Immunopositive cells were counted in 5 randomly selected high-power fields (original magnification, ×40) per biopsy specimen by an observer in a blinded manner. One-way ANOVA was used for analysis of statistical significance.

### Measurement of skin and lung fibrosis.

Dermal thickness was determined at 5 randomly selected sites as previously described (65). Hubner score, reflecting both severity and extent of lung fibrosis, was determined in a blinded manner by a pulmonary pathologist.

### MicroCT of the lung.

Lung CT images were acquired using a Mediso nanoScan8 PET/CT system (Mediso USA) using 50 KeV energy, an exposure time of 300 milliseconds for each projection for a total of 710 projections per mouse. We reconstructed 3D images with a final isotropic spatial resolution of 100 μm. Mice were maintained in a supine position under anesthesia (isofluorane mixed with 100% oxygen) delivered through a nose cone. The 3D images were exported offline in Digital Imaging and Communications in Medicine format and analyzed using threshold segmentation algorithms provided in ITK-SNAP software (67). Varying Hounsfield units (HU) threshold windows were used to differentiate healthy tissue from fibrotic lung tissue. Total lung volume for each mouse was determined using a threshold window that enabled masking of whole lung and removal of surrounding tissue (–1000 to +500 HU). Two thresholds were then used to acquire normal tissue (–1000 to –100 HU) and abnormal or fibrotic tissue (–100 to +500 HU). The 3 sets of volumetric data corresponding to different threshold windows were generated for each of the mice scanned for each cohort. The control group (i.e., mice without fibrosis) was used as a standard reference value to generate the index factors (i.e., the lung FI).

### Flow cytometry.

Flow cytometry analysis of the skin, lungs, and spleen was performed as described (68). Briefly, mice were perfused with cold PBS via cardiac puncture, and organs were collected, triturated, and incubated in digestion buffer (1 mg/mL Collagenase D and 0.1 mg/mL DNase I, both from Roche) for 1 hour at 37°C. Homogenized tissues were passed through 40 μm nylon mesh and washed to obtain single-cell suspensions. The RBCs were lysed using ammonium chloride. Cells were incubated with mouse Fc Block (BD Biosciences; catalog 553142) followed by appropriate Ab cocktails. These Abs included MARCO (clone FAB2956A; R&D Systems), CD45 (clone 30-F11, BD Biosciences), CD11b (clone M1/70, Biolegend), CD11c (clone N418, Biolegend), Ly6C (clone HK1.4, Biolegend), Ly6G (clone 1A8, Biolegend), MHC II (M5/114/15.2, Biolegend), CD80 (clone 16-10A1, Biolegend), CD86 (clone GL-1, Biolegend), B220 (clone RA3-6B2, Biolegend), CD3 (clone 500A2, Biolegend), CD4 (clone RM4-5, Biolegend), CD8 (clone 53-6.7, Biolegend), PDGF-Rα (clone APA5, Biolegend), CD25 (clone 3C7, Biolegend), FoxP3 (clone FJK-16s, Biolegend), IL-6 (clone MP5-20F3, Biolegend), IL-12 (clone c17.8, Biolegend), Smad2/Smad3 (clone O72-670, Biolegend), pSTAT1 (clone 4a, Biolegend), pSTAT3 (clone 4/P-STAT3, Biolegend), pSTAT5 (clone 47/STAT5 pY694, Biolegend) iNOS (clone 25-5920-80, eBioscience), CD206 (clone 141708, eBioscience), CD90.2 (clone 30-H12, Biolegend), EgR2 (clone 12-6691-82, eBioscience), CD38 (clone 90, Biolegend), NK1.1 (clone PK136, Biolegend), CD62L (clone MEL-14, Biolegend), CD44 (clone IM7, Biolegend), CD28 (clone 37.51, Biolegend), and SiglecF (clone E50-2440, BD Biosciences). Live/dead fixable blue (Invitrogen, Carlsbad, CA; catalog L-23105) was added to the cells thereafter to label dead cells prior to cell fixation with 0.5% formaldehyde diluted with PBS. Each incubation step was performed at 4°C for 30 minutes in the dark. A 6-laser Fortessa flow cytometer (BD Biosciences) was used to enumerate cell populations, and the data were analyzed using FlowJo software (TreeStar, Ashland, OR). For ex vivo differentiation assays, bone marrow–derived macrophages were fixed and permeabilized using the FoxP3 staining buffer kits (eBioscience) and immunostained with Abs to iNOS, EgR2, and CD206. Cells were acquired on a BD Fortessa flow cytometer and analyzed using BD FlowJo, version 9. As controls, fluorescence minus one was used to place the gates for analysis.

### Statistics.

Data are presented as mean ± SEM. Two-tailed Student’s *t* test or Mann Whitney *U* test was used for comparisons between 2 groups. If an experiment involved more than 3 groups, 1-way ANOVA followed by Tukey’s or Šidák’s analysis was used to examine for statistical significance. The χ^2^ analysis was used for categorical variables. Statistical significance was set at *P* < 0.05. Pearson’s correlation for continuous variables and Spearman’s correlation for ordinal variables were used to evaluate relationships between 2 variables. Data were analyzed and graphs were created using GraphPad Prism, version 7.03.

### Study approval.

All animal studies were conducted in accordance with NIH guidelines for the care and use of laboratory animals, and protocols were approved by the IACUC of Northwestern University. Studies involving human participants were approved by the IRB of Northwestern University, and all participants provided written informed consent.

## Author contributions

DX, S Bhattacharyya, SDM, and JV formed the hypothesis, designed all experiments, analyzed the results, and prepared the manuscript. DX, WW, MYACW, S Bale, and RGM performed the in vivo experiments. DX and WW conducted the in vitro experiments. DX and S Bhattacharyya analyzed and interpreted the data. DX, S Bhattacharyya, and DG analyzed tissue morphology and expression levels. AY determined lung fibrosis score. DP performed lung CT imaging and data analysis. All authors contributed to the preparation of the manuscript.

## Supplementary Material

Supplemental data

## Version 1 02/01/2022

In-Press Preview

## Version 2 03/08/2022

Electronic publication
