# Supplementary material for: PLG nanoparticles target fibroblasts and MARCO+ monocytes to reverse multiorgan fibrosis
Source: JCI Insight. 2022 Mar 8;7(5):e151037. doi: 10.1172/jci.insight.151037 (PMC8983146; doi:10.1172/jci.insight.151037)
Supplement: Supplemental data [file jciinsight-7-151037-s140.pdf]

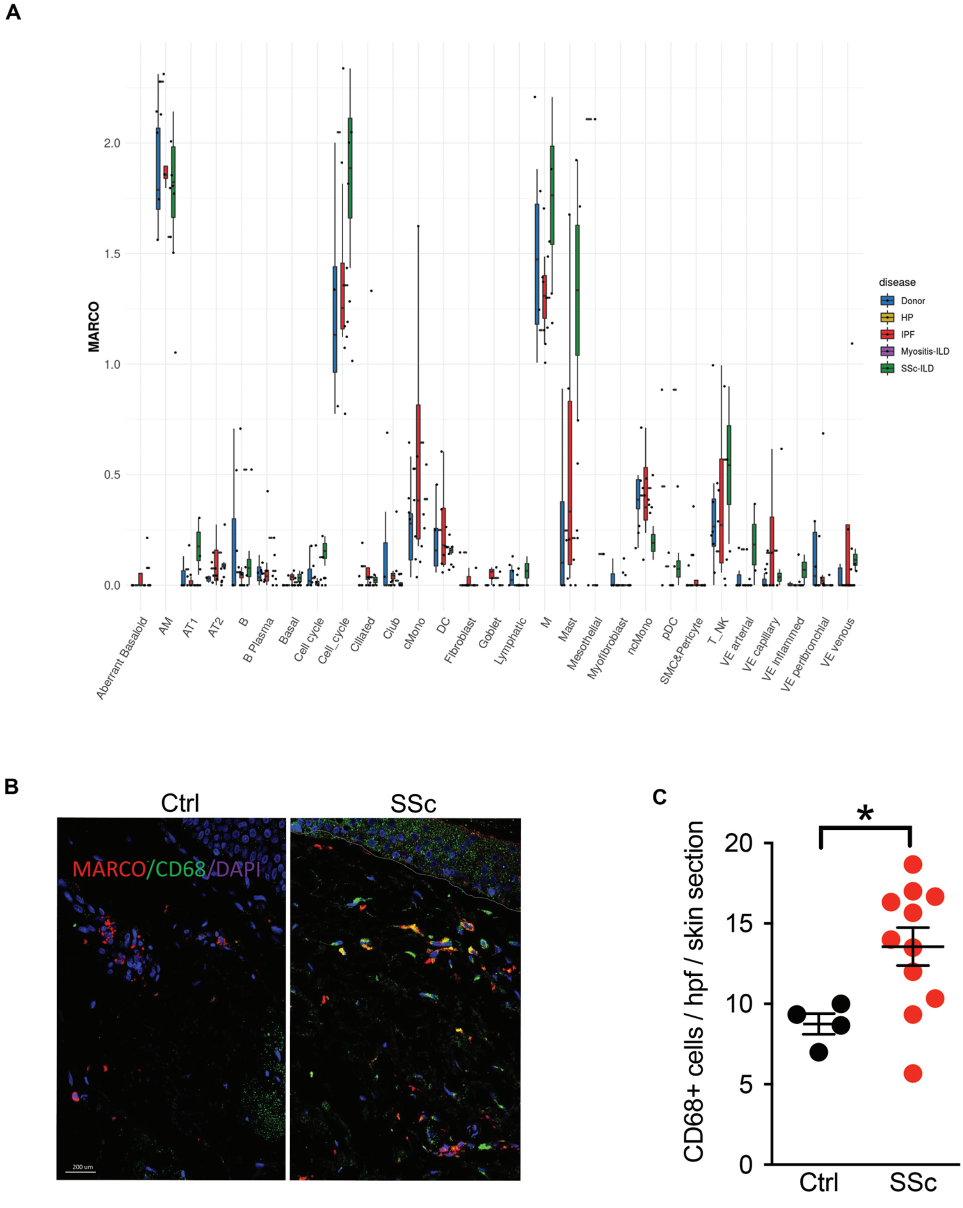

A
MARCO
2.0
1.5
1.0
0.5
0.0
disease
Donor
HP
IPF
Myositis-ILD
SSc-ILD
Aberrant Basaloid
AM
AT1
AT2
B
B Plasma
Basal
Cell cycle
Cell_cycle
Ciliated
Club
cMono
DC
Fibroblast
Goblet
Lymphatic
M
Mast
Mesothelial
Myofibroblast
ncMono
pDC
SMC&Pericyte
T_NK
VE arterial
VE capillary
VE inflammed
VE peribronchial
VE venous
B
Ctrl
SSc
MARCO/CD68/DAPI
200 um
C
*
CD68+ cells / hpf / skin section
20
15
10
5
0
Ctrl
SSc

Supplementary Figure 1. Upregulated MARCO expression in the skin of SSc patients colocalizes with macrophages. (A) Boxplots representing the percent makeup distributions of MARCO expression in different cell types within each disease group of the single-cell RNAseq data from the idiopathic pulmonary fibrosis cell atlas (Misharin) with markedly elevated MARCO cell types highlighted in red. Each dot represents a single subject, whiskers represent 1.5x interquartile range (IQR). (B) Representative confocal immunofluorescent (IF) images of MARCO (red) and CD68 (green) expression in skin biopsies from patients with and controls. Nuclei were identified by DAPI (blue). Scale bar, 200 μm. (C) Dot plots of frequency of CD68+ myeloid cells (mean ± SEM) determined from 5 high-power fields (hpfs) per section in each biopsy specimen.

Supplementary Figure 2

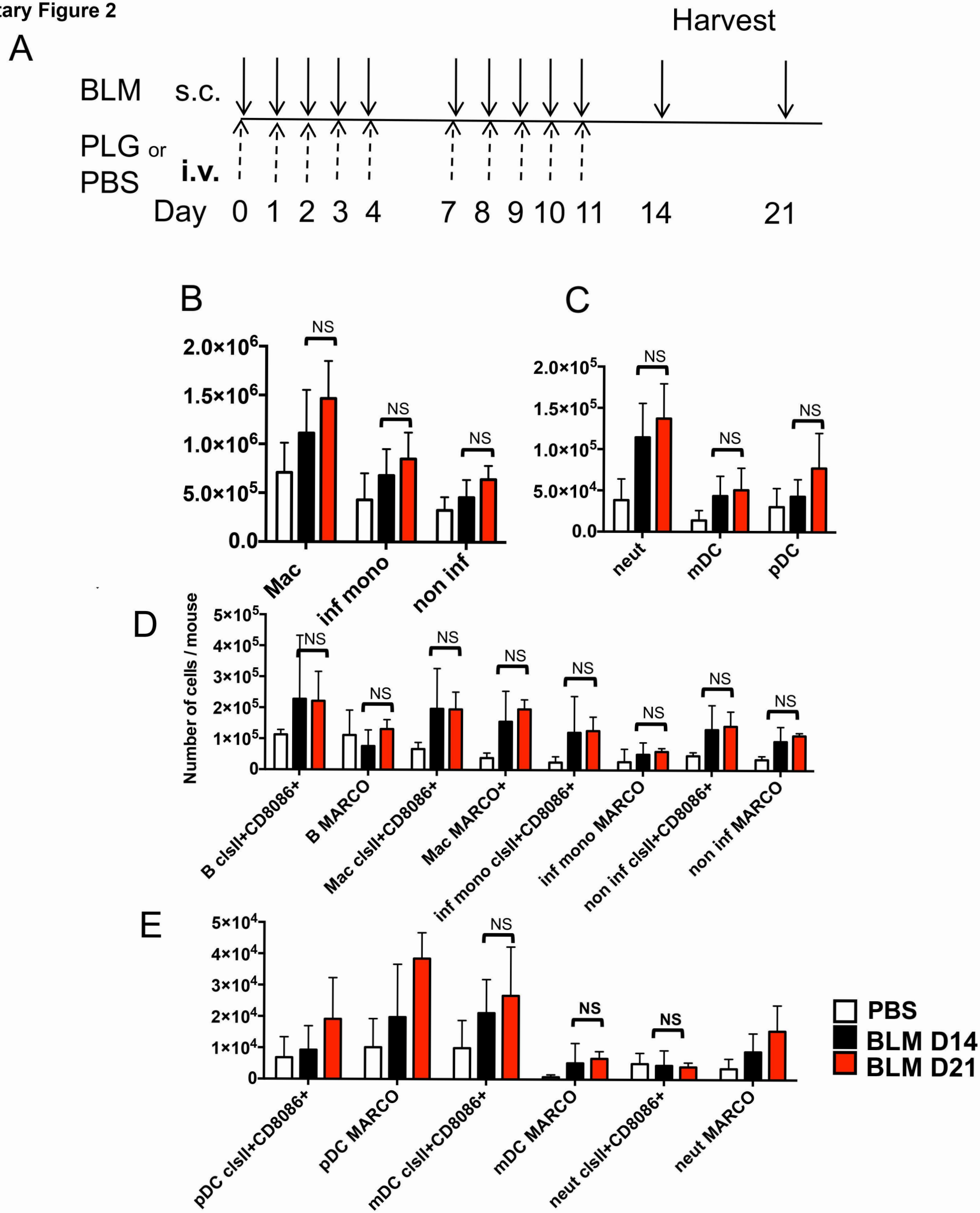

Supplementary Figure 2. BLM induced persistent inflammation in the mouse lung. (A) Schema of experimental setup and treatment regimen. (B and C) Total number of myeloid subset infiltrates, including inflammatory monocytes (inf mono), macrophages (mac), myeloid dendritic cells (mDC), and non-inflammatory monocytes (non-inf mono), in the lung at day14 (D14) and day 21 (D21) post BLM injection was determined by flow cytometric analysis. (D and E) Quantitation of differentially activated myeloid cell subsets was measured by expression of MHC II (clsII), CD80, and MARCO. All graphs were generated with n=4-5 mice/group (3 independent repeats) and show means $\pm$ SEM. NS, not significant via one-way analysis of variance followed by Sidak's multiple comparison test.

Supplementary Figure 3

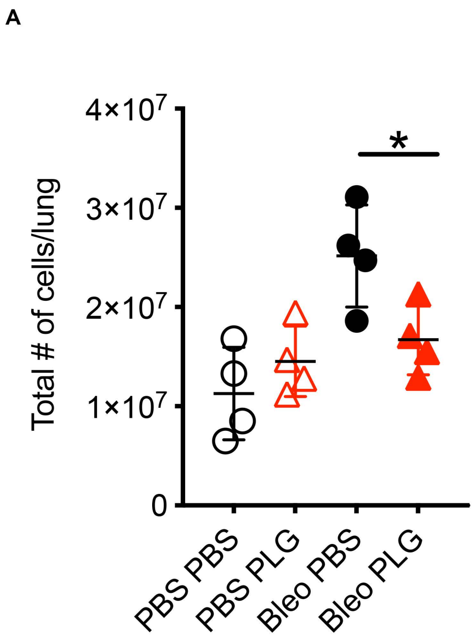

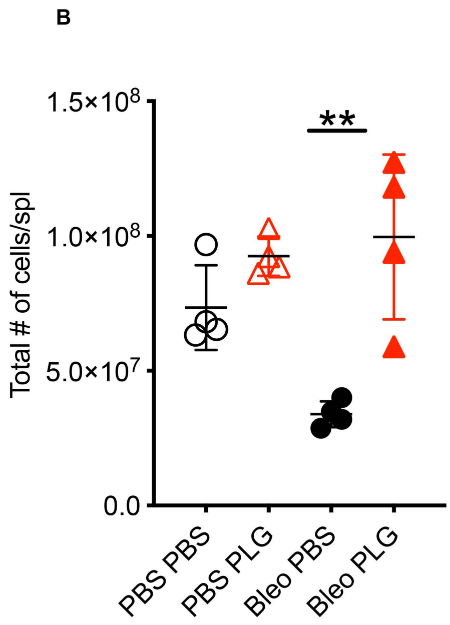

Supplementary Figure 3. PLG nanoparticles significantly reduced leukocytes in the lungs while maintaining splenocyte count in BLM-injected mice. Total number of leukocytes in the lung (A) and spleen (B) at day14 (D14) post BLM injection was measured by flow cytometric analysis. All graphs were generated with n=4-5 mice/group for at least 3 independent repeats and show means ± SEM. *p<0.05 and **p<0.01 via one-way analysis of variance followed by Sidak's multiple comparison test.

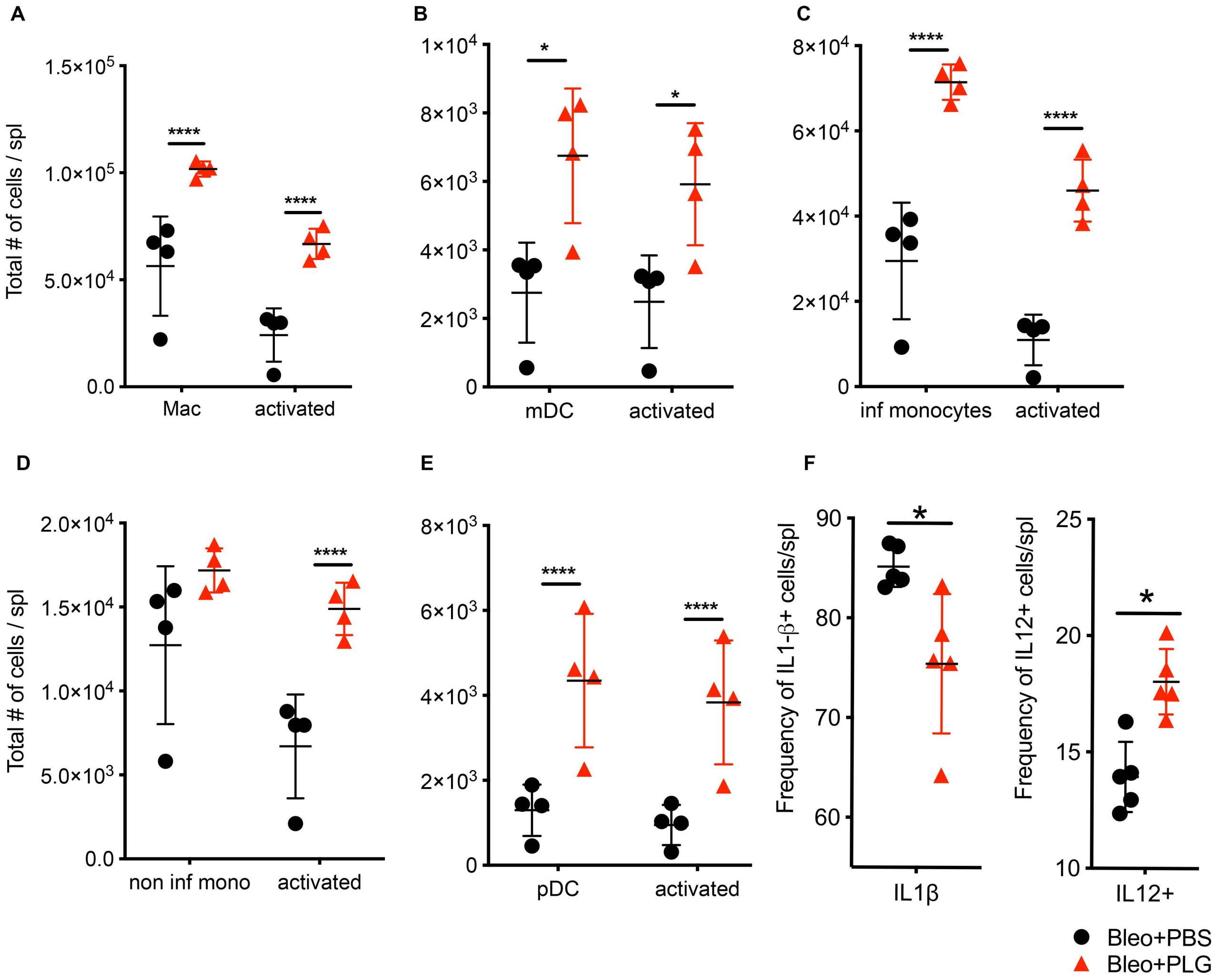

A
B
C
D
E
F
Total # of cells / spl
1.5×10^5
1.0×10^5
5.0×10^4
0.0
****
****
Mac
activated
1×10^4
8×10^3
6×10^3
4×10^3
2×10^3
0
*
*
mDC
activated
8×10^4
6×10^4
4×10^4
2×10^4
0
****
****
inf monocytes
activated
2.0×10^4
1.5×10^4
1.0×10^4
5.0×10^3
0.0
****
non inf mono
activated
8×10^3
6×10^3
4×10^3
2×10^3
0
****
****
pDC
activated
Frequency of IL1-β+ cells/spl
90
80
70
60
*
IL1β
Frequency of IL12+ cells/spl
25
20
15
10
*
IL12+
Bleo+PBS
Bleo+PLG

Supplementary Figure 4 PLG nanoparticles significantly sequestered leukocytes in the spleen and skewed CD4+T cells towards a Th1 phenotype in BLM-injected mice. Total numbers of normal and activated myeloid cells (A-E) and cytokine-producing leukocytes (F) at day14 (D14) post BLM injection was measured by flow cytometric analysis employing intracellular cytokine staining. All graphs were generated with n=4-5 mice/group for at least 3 independent repeats and show means $\pm$ SEM. *$p<0.05$ and ****$p<0.0001$ via one-way analysis of variance followed by Sidak's multiple comparison test.
